# Inflammation at the maternal-fetal interface: a perspective on interacting risk factors for preterm birth in sub-Saharan African women living with HIV

**DOI:** 10.3389/fimmu.2026.1741921

**Published:** 2026-03-16

**Authors:** Jasmine S. Edwards, Kristina De Paris

**Affiliations:** Department of Microbiology and Immunology, University of North Carolina, Chapel Hill, NC, United States

**Keywords:** HIV, inflammation, macrophage function in pregnancy, preterm birth, vaginal microbiota

## Abstract

Globally, approximately 10% of all babies are born prematurely. The vast majority of preterm births, defined as birth <37 weeks of gestation, occur in low- and middle-income countries (LMICs) in Asia and Africa. Furthermore, premature birth has become the leading cause of death in infants under the age of 5 years. Thus, to improve maternal and infant health outcomes, better diagnostics and intervention strategies are urgently needed. However, the multifactorial etiology of preterm birth provides a major obstacle in achieving this goal. A common factor to many adverse birth outcomes, including preterm birth, is aberrant immune activation at the maternal-fetal interface. The specific cause of immune activation, however, remains unknown. Both HIV and an anaerobe-rich vaginal microbiota have been independently identified as risk factors for preterm birth, and both factors also promote inflammation and immune activation at mucosal sites. The interplay of HIV and microbiota is widely acknowledged, although mostly in the context of the intestinal microbiome. This review will highlight how the regulatory function of macrophages at the maternal-fetal interface can be altered in response to HIV and antiretroviral therapy and to changes in vaginal microbiota. We proceed to discuss interactions between the various factors and propose a dual-hit model in which macrophages act as mediators of inflammation at the maternal-fetal interface in response to specific vaginal commensals and HIV infection in sub-Saharan African women with preterm birth outcomes.

## 1 Introduction

Globally, in 2020, 13.4 million babies were born prematurely, corresponding to 1 in 10 births (1). Preterm birth represents an important health problem as it is associated with both maternal and infant morbidity and mortality. In the decade from 2010 to 2020, preterm birth rates have remained stagnant (2, 3). Preterm birth can occur spontaneously or be provider-initiated. It is estimated that 40-75% of all preterm births occur spontaneously (4–6). Spontaneous preterm birth (sPTB) is defined as early delivery initiated by the rupture of the fetal membranes and/or onset of uterine contractions (early labor).

The etiology of preterm birth is multifactorial. South Asia and sub-Saharan Africa account for the vast majority of preterm births worldwide (2, 3, 7). Limited healthcare infrastructure in low- and middle-income countries (LMICs) of these regions likely contributes to this high burden, but many other factors impact pregnancy outcomes. Increased maternal age, chronic stress, comorbidities (e.g., infectious diseases, diabetes, hypertension), environmental factors, and racial disparities have all been associated with a higher risk for preterm birth (2, 3). Inflammation, a factor common to many of these conditions, has also been implicated in preterm birth (4, 8). The source of inflammation, however, often remains unknown. Thus, to reduce the global burden and impact of preterm birth on maternal and child health, a deeper understanding of biological mechanisms promoting spontaneous preterm birth is needed to develop novel diagnostic and intervention tools.

This review will focus on the role of macrophages in pregnancy and how altered macrophage function at the maternal-fetal interface could promote preterm birth. Recent studies of adverse pregnancy outcomes have highlighted the potential role of vaginal microbiota on pregnancy outcomes. Therefore, we will also discuss the available knowledge on the vaginal microbiome in the context of preterm birth and aim to establish potential links between specific vaginal microbiota and macrophage function in pregnancy. Furthermore, considering that sub-Saharan Africa has some of the highest preterm birth rates and represents a region with a high incidence of HIV, we will outline how HIV infection and antiretroviral therapy (ART) may impact preterm birth outcomes. Throughout, we will emphasize known and potential interactions between macrophages and microbiota, macrophages and HIV/ART, and HIV/ART and microbiota. Finally, we will propose a dual-hit model to explain the increased risk of preterm birth in pregnant sub-Saharan African women with HIV. The dual-hit model proposes that macrophages act as mediators of inflammation in response to both anaerobic vaginal commensals and HIV. Consequently, macrophages will maintain a more persistent bias towards inflammatory function throughout pregnancy. Interference with the regulatory function of macrophages at the maternal-fetal interface may promote early labor and preterm birth. Yet many open questions remain, and the review will conclude with some of these questions and challenges in answering them.

## 2 The role of macrophages in pregnancy

### 2.1 Pregnancy overview

Successful progression of pregnancy to full-term birth requires tightly regulated interactions between sex hormones, host immunity, and the microbiome in the female genital tract. From implantation to labor, the highly synchronized immune system facilitates a successful term delivery at or after 37 weeks of gestation through tightly regulated shifts in the immune milieu at the maternal-fetal interface. The maternal-fetal interface consists of the maternal-derived decidua and fetal-derived placenta. Its milieu is therefore regulated by both maternal and infant factors and their interactions. Immune cell populations at the maternal-fetal interface undergo dynamic changes during pregnancy that are reflected in shifts of relative frequencies and functions of specific cell populations in the various trimesters. This review will focus specifically on the role of macrophages. The proposed functions of macrophages during pregnancy are primarily based on mouse models or human post-delivery blood and tissue samples from the Female Reproductive Tract (FRT).

### 2.2 Macrophage polarization

Macrophages can be categorized into two main subsets, M1 and M2 macrophages (**Table 1**). In general, M1 macrophages are involved in inflammatory responses, whereas M2 macrophages exert immunoregulatory functions. Macrophage polarization is not a finite state and can shift between M1 and M2 states (9). It depends on local environmental cues, such as the direct interactions of macrophages with other cells or responses to local soluble mediators (e.g., cytokines). The activation of macrophages is often initiated by Pathogen- or Damage-Associated Molecular Patterns (P/DAMPs) of microbes or danger signals that engage their relevant Pattern Recognition Receptors (PPRs) on macrophages.

**Table 1:** Human macrophage polarization, *in vitro*.

Macrophage subset	M1	M2a	M2b	M2c	M2d
Inducing Factors	GMCSF, IFNγ, Bacteria, Viruses	M-CSF, IL-4, IL-13	TLR ligands, IL-1β	IL-10, TGFβ	IL-6, TLR agonists
Cell Surface Markers	CD14, CD64, CD68, CD80/86, HLA-DR, TLR2, TLR4	CD14, CD68, CD206	CD14, CD68, CD86	CD14, CD68, CD163, CD206, TLR1, TLR8	CD14, CD68, IL-6R, TLRs
Transcription Factors	STAT1, STAT5, NFκB, IRF3, IRF5	STAT3, STAT6, IRF4, PPARγ, PPARδ	STAT3, STAT6, IRF4, PPARγ, PPARδ	STAT3, STAT6, IRF4, PPARγ, PPARδ	STAT3, STAT6, IRF4, PPARγ, PPARδ
Intracellular Markers	IL-1α, IL-1β, TNF-α, IL-6, IL-12, IL-23, iNOS	TGF-β, IL-1ra, IL-6, IL-10, IL-12, IL-23, Arg1	TNF-α, IL-1, IL-6, IL-10	TGF-β, IL-10, Arg1	TGF-β, TNF-α, IL-10, IL-12
Function	Proinflammatory Activity, Tissue Damage, Anti-Microbial Activity	Endocytosis, Cell Growth, Tissue Repair, Immunosupression	T_H_2 Differentiation, Promote Microbial Infections	Phagocytosis of Apoptotic Cells, Immunosupression	Angiogenesis, Tumor Growth

*In vitro*, macrophages can be polarized into M1 or M2 macrophages by stimulation with granulocyte/monocyte colony stimulating factor (GM-CSF), followed by lipopolysaccharide (LPS) and interferon gamma (IFN-γ) stimulation, or by monocyte colony growth factor (M-CSF) followed by interleukin 4 (IL-4) stimulation, respectively (10). Macrophage polarization is tightly linked to arginine metabolism. M1 macrophages express inducible nitric oxide synthase (iNOS) that converts arginine to nitric oxide (NO) and reactive oxygen species (ROS), whereas M2 macrophages use arginase to hydrolyze arginine to urea and ornithine (11). The activation of the nuclear factor kappa-light-chain-enhancer of activated B cells (NF-kB) pathway and subsequent activation of Signal transducer and activator of transcription 1 (STAT1) will result in the production of proinflammatory cytokines (IL-12, IL-1β, IL-6, IL-23, Tumor Necrosis Factor Alpha [TNF-α]) by M1 macrophages. M2 macrophages activate STAT6 and produce anti-inflammatory cytokines, such as IL-10 and TGF-β (12, 13). M2 macrophages can be further divided into M2a-d subsets (14). M2a macrophages enhance endocytosis and promote cell growth and tissue repair. M2b macrophages, despite producing both anti- and pro-inflammatory cytokines, promote the differentiation of CD4^+^ T cells to T_H_2 cells and aid in the phagocytosis and clearance of parasites, bacteria, and fungi. M2c macrophages are primarily activated following anti-inflammatory responses as phagocytes for apoptotic cells. M2d macrophages, often referred to as tumor-associated macrophages, function in tumorigenesis and promote angiogenesis and tumor growth (15–18).

### 2.3 Macrophage function in normal pregnancy with term and live birth outcome

Macrophages represent between 20% to 30% of decidual leukocytes, a percentage much higher than the frequency of monocytes in peripheral blood. Prior review papers proposed that during implantation, Natural Killer (NK) cells and inflammatory M1-like macrophages migrate into the placenta and secrete cytokines and growth factors that are necessary to support placental remodeling and placental vascularization (19–24). Specifically, macrophages secrete growth factors and cytokines (e.g., IL-12, IL-1β, TNF-α, IL-6, and NO) (19), consistent with an M1-like macrophage function. *In vitro* studies determined that an increased production of vascular endothelial growth factor (VEGF), a crucial growth factor for placental development (25, 26), promotes M2 macrophage polarization and recruitment (27). Tissue remodeling and angiogenesis, including spinal artery formation, ensure adequate blood and nutrient transfer from mother to infant (28). Once trophoblasts start to invade the uterine stroma, a transition from M1 to immunoregulatory M2 macrophages occurs to ensure the development of the allogenic fetus (29–32).

Heikkinen et al. characterized human decidual macrophages, *in vitro*, from pregnant women who delivered at term. Protein analysis of decidual macrophages confirmed their M2 bias, as the expression of CD80 and CD86 was reduced (33). Interestingly, decidual macrophages spontaneously produce low levels of IL-10, which increases following exposure to Lipopolysaccharide (LPS), while the production of TNF-α and IL-1β in response to LPS decreases. Furthermore, decidual macrophages produce high levels of tryptophan catabolizing enzyme indoleamine 2,3-dioxygenase (IDO), which, as observed in mice, promotes immunotolerance by suppressing maternal T cell activation against the fetus (34). Utilizing peripheral blood samples from pregnant women at week 28 of gestation and at term, Sykes et al. confirmed that IL-10 is spontaneously produced at week 28 to support an M2-like immunoregulatory state during fetal development, but IL-10 decreases at the time of labor (35). As pointed out in a review by Vishnyakova, the M2-macrophage-associated immunotolerance coincides with a shift in CD4^+^ T cells from T helper 1 (T_H_1) to T_H_2 and from T_H_17 to regulatory T cells (Treg) (36). Costa et al. further defined CD14^+^ myeloid populations of the placenta during fetal development into a predominant subset of IL-10-producing HLA-DR^high^ CD14^+^ myeloid cells (“M2”) and a subset of IL-1β and IL-6 producing HLA-DR^low^ CD14^+^ myeloid cells (“M1”). They also suggested that the presence of programmed cell death 1 (PD1) ligand on myeloid cells and PD1 on CD8^+^ and CD4^+^ T cells of the human placenta may be crucial for maintaining tolerance (37).

During labor, macrophages play a role in stimulating uterine contractions to facilitate the expulsion of the baby and placenta, which is followed by uterine involution. This is initiated by the recruitment of decidual macrophages, decidual NK cells, and T cells to the myometrium. Inflammatory cytokines such as IL-1β, IL-6, and IL-8 were identified in tissue biopsies from humans in fetal membranes, decidua, cervix, and the myometrium before labor (38, 39). Phillippe et al. hypothesized that placental macrophages may be skewed towards M1 due to the possible triggering of TLR9 by cell-free fetal DNA and subsequent production of inflammatory cytokines and chemokines (40). These innate immune responses in the cervix, decidua, myometrium, and chorion lead to the production of uterine-activation proteins. Combined, these processes result in cervical ripening, rupture of the fetal membranes, and phasic myometrial contractions and culminate in parturition.

Right before parturition, based on studies in mice, Shynlova et al. proposed that a wave of decidual macrophages floods the myometrium with proinflammatory signals, although anti-inflammatory cytokines such as IL-10 and TGF-β1 are also detected. This may be a mechanism to help regulate the inflammatory state of childbirth (41). Furthermore, it is suggested that the M2-like fetal macrophages called Hofbauer cells (HBCs) are involved in parturition by the secretion of C-C motif chemokine ligand (CCL) 2, CCL13, CCL14, CD209, and annexin 2 (A2) (42). Kim et al. compared maternal decidual macrophages with fetal HBCs obtained from the placenta after delivery. For both, DNA methylation profiling revealed hypomethylation for M2-specific genes (e.g., CCL13, CCL14, Alpha-2-Macroglobulin (A2M), histamine N-methyltransferase (HNMT), and IL10) and production of sustained levels of IL-10, TGF-β, and Arg (42). In contrast, M1 genes (e.g., Toll-Like Receptor (TLR) 9, IL-1β, IL12 receptor Beta 2 subunit (IL12RB2), cluster of differentiation (CD) 48, and Fes-related tyrosine kinase gene (FGR-associated with fetal growth restriction) are hypermethylated to support the tolerogenic milieu at the maternal-fetal interface following labor. Other studies also observed significantly higher levels of IL-6 compared to IL-10 when analyzing fetal macrophages (or Hofbauer cells, HBC) (43).

**Figure 1:**
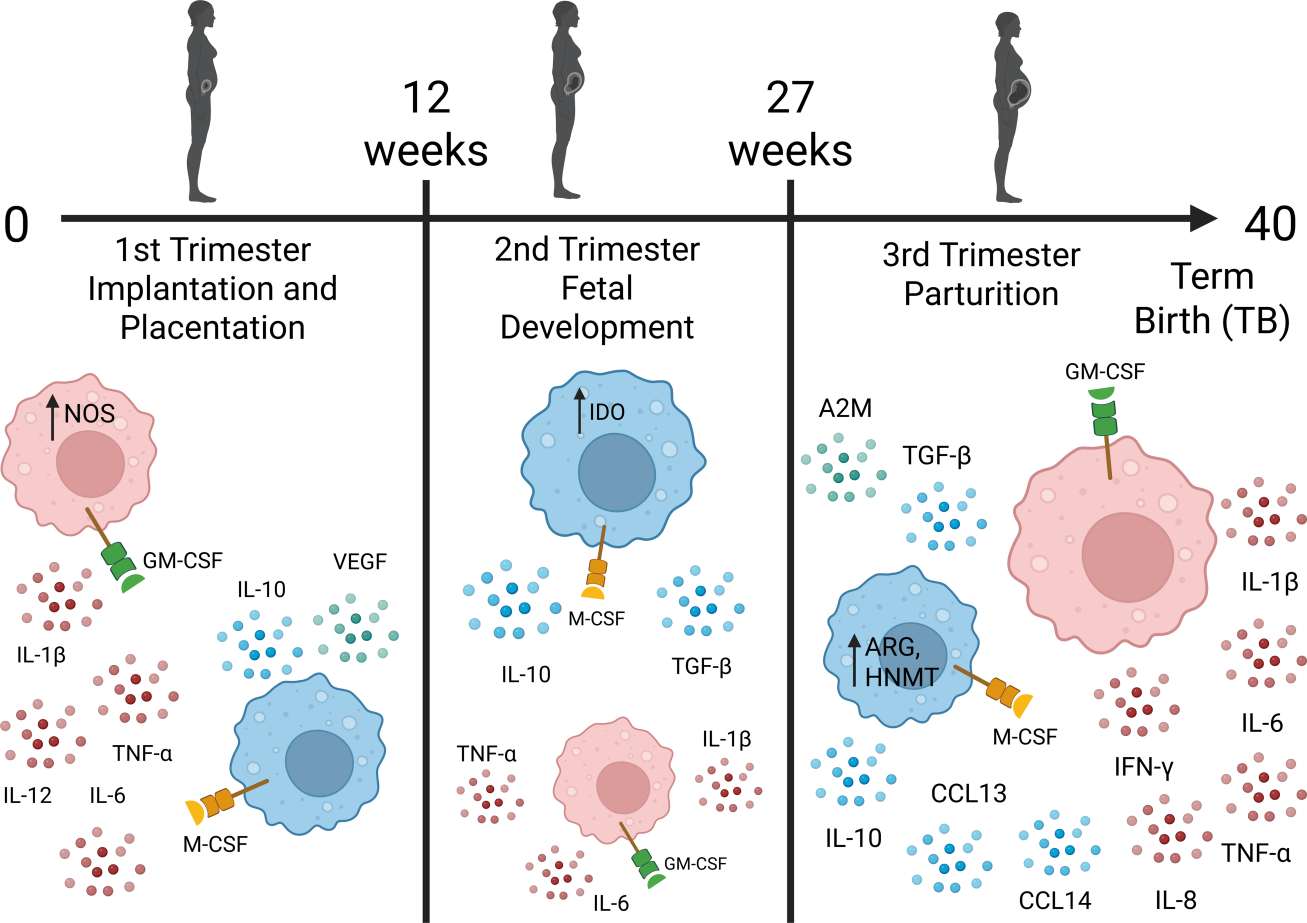
Proposed shifts in maternal decidua macrophage populations during healthy pregnancy. In the 1^st^ trimester, implantation is characterized as an inflammatory process dominated by M1 macrophages (red). Upon embryo attachment to the uterine wall, there is a shift to a mixture of M1/M2 macrophages that facilitate placentation. The 2^nd^ trimester comprises most of the fetal development stage in which M2 macrophages (blue) dominate the microenvironment of the maternal-fetal interface to promote tolerance of the fetus and paternal antigens. During the 3^rd^ trimester, there is a shift towards an inflammatory microenvironment dominated by M1 macrophages to induce labor at term (37-40 gestational weeks). During labor, a rush of M2 macrophages occurs to initiate tissue repair post-partum. Please see text for details and references (section 1.3). Created in BioRender. Edwards, J. (2026) https://BioRender.com/57tn9qd.

A summary of proposed maternal (decidua) macrophage populations for women with term birth is represented in **Figure 1**. Overall, there is evidence from humans and mouse models that maternal decidua macrophages and fetal macrophages (HBCs) are skewed towards the M2 phenotype during fetal development and following labor. This suggests that M2 macrophages are crucial in promoting immunotolerance to the fetus during development and healing of the FRT following labor. In contrast, M1 macrophages are crucial for implantation, placental development, and initiating labor.

### 2.4 Altered macrophage function in pregnancies with adverse outcomes

PTB has been associated with increased numbers of macrophages, but it remains unknown whether there is a dysregulation in the coordinated macrophage polarization shifts seen in term birth. Imbalances of M1 and M2 polarization during early or late pregnancy have been linked to adverse birth outcomes (44). In early pregnancy, uncontrolled inflammation is associated with early-onset preeclampsia and spontaneous abortions.

Utilizing a mouse model of pregnancy, one study attributed spontaneous abortion to a significant decrease in placental vacuolar (V) ATPase (a2V) (45). V-ATPase is important in macrophage polarization by inducing monocyte chemoattractant protein (MCP-1) that modulates macrophage influx and trophoblast invasion in early pregnancy. Similarly, macrophage migration inhibitory factor (MIF) is key to facilitating embryo implantation and pregnancy maintenance (46, 47). In early gestation, when inflammation aids in implantation and placentation, decreased MIF levels are linked with miscarriage. In contrast, increased MIF levels in mid-gestation, when immunotolerance is needed for proper fetal development, are associated with preterm birth and preeclampsia (48–50). Elevated levels of TNF-α and activated M1 macrophages in the decidua in mid-gestation are similarly associated with preterm birth and preeclampsia (51–53). *In vitro*, TNF-α has been shown to induce Fas Ligand (FasL) on human decidual M1 macrophages and to promote trophoblast apoptosis (51–53).

Inflammation has been indicated as a key factor in spontaneous preterm birth. As macrophages play a key immunomodulatory role at the maternal-fetal interface, a shift towards M1 polarization during the second and third trimesters could potentially impact preterm birth. Indeed, in mice, LPS treatment promotes M1 polarization and preterm birth (54). M1 polarization was mediated via Neurogenic locus notch homolog protein 1 (Notch-1) signaling (54, 55). Similarly, increased frequencies of M1 macrophages that were accompanied by elevated levels of the proinflammatory cytokines IL-6 and IFN-γ were observed in a different study when mice with preterm birth were compared to mice with term birth (56). In yet another mouse study, complement protein 5a (C5a) was demonstrated to induce production of matrix metalloproteinases (MMP), especially MMP9, by M1 macrophages that then promote collagen degradation in the cervix and cervical ripening in mice with preterm birth, whereas MMP9 in term birth was predominantly derived from cervical fibroblasts and epithelial cells (57).

**Figure 2:**
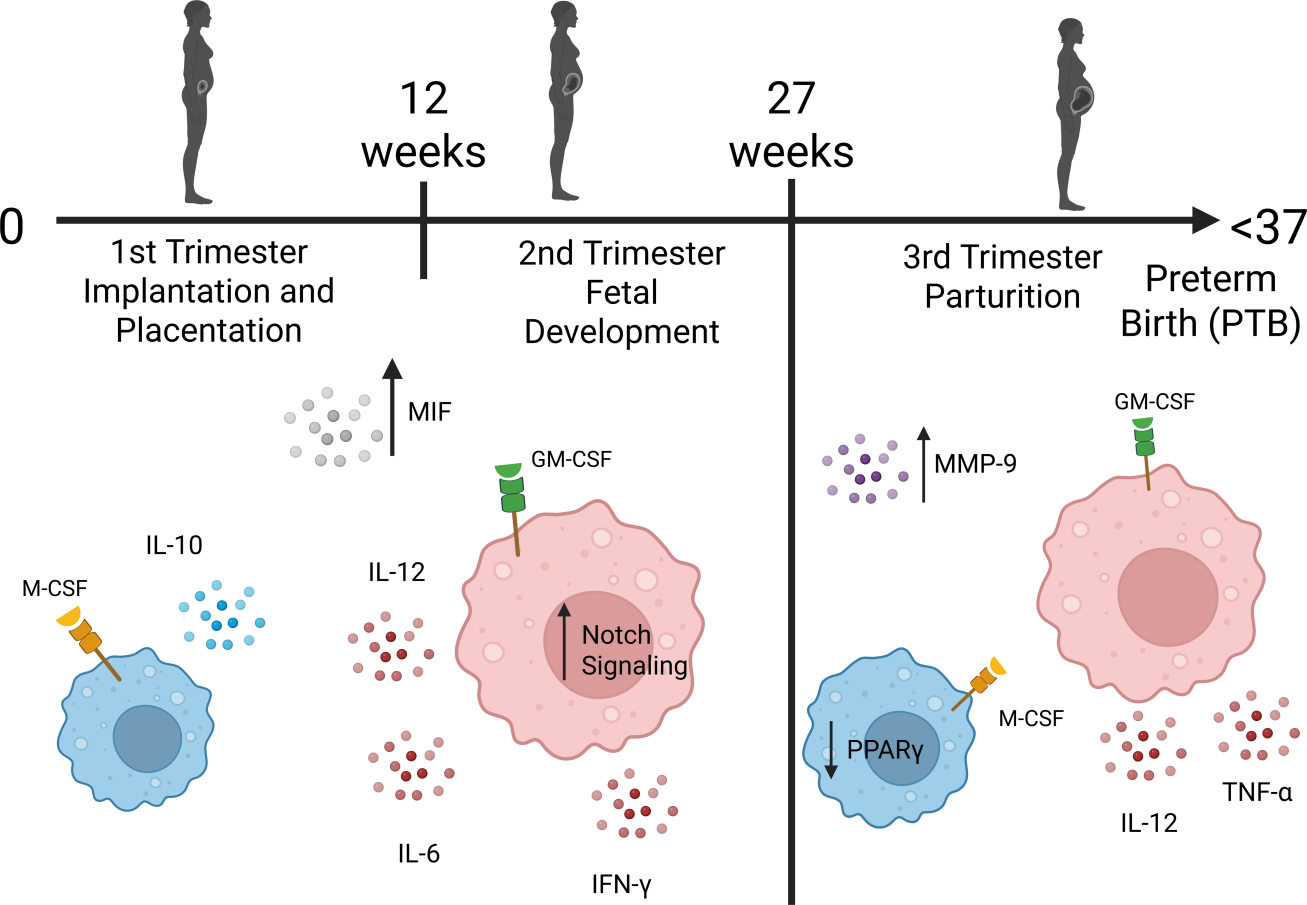
Proposed shifts in maternal decidua macrophage populations during complicated pregnancy. In early-middle pregnancy (late 1^st^ trimester/2^nd^ trimester) there is a mixture of M1 (red) and M2 (blue) macrophages. The increased expression of macrophage migration inhibitory factor (MIF) is a marker for inflammation as it encourages (M1) macrophages to remain at the site of “infection” or “injury” while inhibiting the influx of peripheral leukocytes. In the decidua, Notch signaling promotes M1 polarization and the secretion of pro-inflammatory cytokines IL-6, IL-12 and IFN-γ. Despite the presence of M2 macrophages secreting IL-10, the immunotolerant M2 dominant microenvironment typical for term pregnancy is disrupted. Furthermore, in late pregnancy, increased production of matrix metalloproteinase 9 (MMP-9) by macrophages (typically secreted from cervical fibroblasts and columnar epithelial cells during term pregnancy) contributes to collagen degradation in the cervix and results in labor initiation. Although M2 macrophages are present, decreased expression of PPARγ results in a decrease in anti-inflammatory cytokine secretion. Overall, M1 macrophage dominance promotes inflammation leading to preterm birth/labor. Please see text for details and references (section 1.4). Created in BioRender. Edwards, J. (2026) https://BioRender.com/8bxp2n7.

While access to relevant tissues from pregnant women hampers validation of these findings in humans, higher frequencies of M1 macrophages have been observed in the decidua and cervix (collected at delivery) of women with preterm birth compared to women with term birth (58). Women with preterm birth also have higher levels of proinflammatory cytokines TNF-α and IL-12 compared to women with term birth. Complementary to this finding, the expression of the transcription factor Peroxisome Proliferator-Activated Receptor gamma (PPARγ), promoting M2 polarization, is reduced (59). A summary of proposed maternal (decidua) macrophage populations for women with preterm birth is represented in **Figure 2**.

Although not the focus of this review, it is important to note that fetal sex differences have been associated with different placental (particularly fetal macrophage) responses. Placentas of male fetuses have a higher expression of TLR4 and greater production of TNF-α compared to placentas of female fetuses (60). Female placentas appear to mount more immunoregulatory and broad antibody responses to stimuli (bacteria, viruses, etc.), whereas male placentas have a rapid and greater inflammatory response (61–63). In the placental villi, HBCs from female fetuses are prone to develop a M2 phenotype compared to HBCs from male fetuses (64). Female HBCs have a higher lipid metabolism, which promotes an immunoregulatory response (64). In contrast, male HBCs exhibit higher glycolytic metabolism and ROS production, both contributing to a rapid inflammatory response (64). During infection, female HBCs will favor a Type I IFN(α) response (for swift clearance of pathogens), whereas male HBCs will favor a Type II IFN(γ) response (65, 66). HBC polarization in response to infection, however, can be variable (67). Some HBCs, independent of fetal sex, will polarize towards M1 macrophages and increase the M1/M2 ratio, whereas some HBCs will retain a M2 phenotype despite producing TNF-α or IFN-γ (67). Although male fetuses are associated with prolonged gestation (68) most preterm-born infants are males. One could speculate that the more inflammatory response of HBCs of male fetuses is a factor in the higher rate of male infants in preterm birth.

Our understanding of human macrophage populations and the timing of coordinated shifts between M1/M2 subsets is limited, as the maternal-fetal interface cannot be accessed during pregnancy. Human samples are limited to pre-labor peripheral blood and post-labor maternal-fetal macrophages from various tissues of the FRT. Although evidence suggests M1 macrophage-associated inflammation contributes to adverse birth outcomes such as preterm birth in both mice and humans, differences in the physiology of mice (e.g., shorter gestational period, litters, postnatal developments, placental structural and function, hormonal shifts) (69–71) may limit the translation of mouse pregnancy data to human pregnancy. Alternative animal models include rabbits, guinea pigs, chinchillas, mouse lemurs, and marmosets (72). The various animal models can inform about immune responses and their relation to macrophage polarization shifts during pregnancy, an important first step in understanding normal and complicated pregnancies in humans. Such data can then be validated by human studies. Furthermore, factors other than macrophage function need to be evaluated for their potential in modulating immune responses at the maternal-fetal interface and birth outcomes.

## 3 The impact of vaginal microbiota on pregnancy outcomes

### 3.1 Overview of the vaginal microbiome

Humans are constantly exposed to potentially pathogenic microbes. At the same time, there are non-pathogenic microbes that are part of the body’s natural flora and have a symbiotic relationship with the human host. The Human Microbiome Project (HMP) was initially created (73) to identify the specific microbial composition at distinct sites (74). Since then, the Integrative Human Microbiome Project emerged with the intent of defining the specific interactions of microbiota at distinct anatomic sites with human health and disease. In the context of pregnancy, the flora of the human FRT (the vagina, cervix, uterus, fallopian tubes, and ovaries) is of particular interest. Controversy remains as to whether the uterus represents a sterile environment (75–78). In healthy women, there is consensus that the upper FRT is much more sparsely populated compared to the lower FRT (79, 80). Therefore, this review will focus on the impact of the vaginal microbiota, and specifically the bacterial flora, on pregnancy outcomes.

The vaginal microbiota is highly diverse; yet the analysis of close to 400 healthy North American women revealed that five main bacterial community states (CST) dominate the vaginal microbiome of most women (81). CSTI is dominated by *Lactobacillus crispatus (L. crispatus)*, CSTII by *Lactobacillus gasseri (L. gasseri)*, CSTIII by *Lactobacillus iners (L. iners)*, CSTIV represents a mixture of strict and facultative anaerobes (Gardnerella, Atopobium, Mobiluncus, Prevotella) with a smaller portion of lactobacilli, and CSTV is dominated by *Lactobacillus jensenii (L. jensenii)*. A key finding of the former study was that differences in the relative percentages of the distinct CSTs in the vaginal microbiota were associated with distinct ethnicities. Specifically, CSTI (*L. crispatus*) was most prevalent in White women (n= 97), CSTIII (*L. iners*) in Asian women (n= 96), both CSTIII (*L. iners*) and CSTIV dominated in Hispanic women (n = 97), and CSTIV in Black women (n= 104). Other studies confirmed that CSTIV was the most common CST in Black American, sub-Saharan African, and Afro-Caribbean women (82–84). As it is generally assumed that lactobacilli are essential for vaginal health (85) whereas other strict anaerobe bacteria promote bacterial vaginosis (BV), it appears imperative to define the impact of these fundamental differences in the composition of the vaginal microbiota in women of distinct ethnicity on health and disease, or, in this review, on pregnancy.

### 3.2 The contributions of Lactobacilli to vaginal health

**Figure 3:**
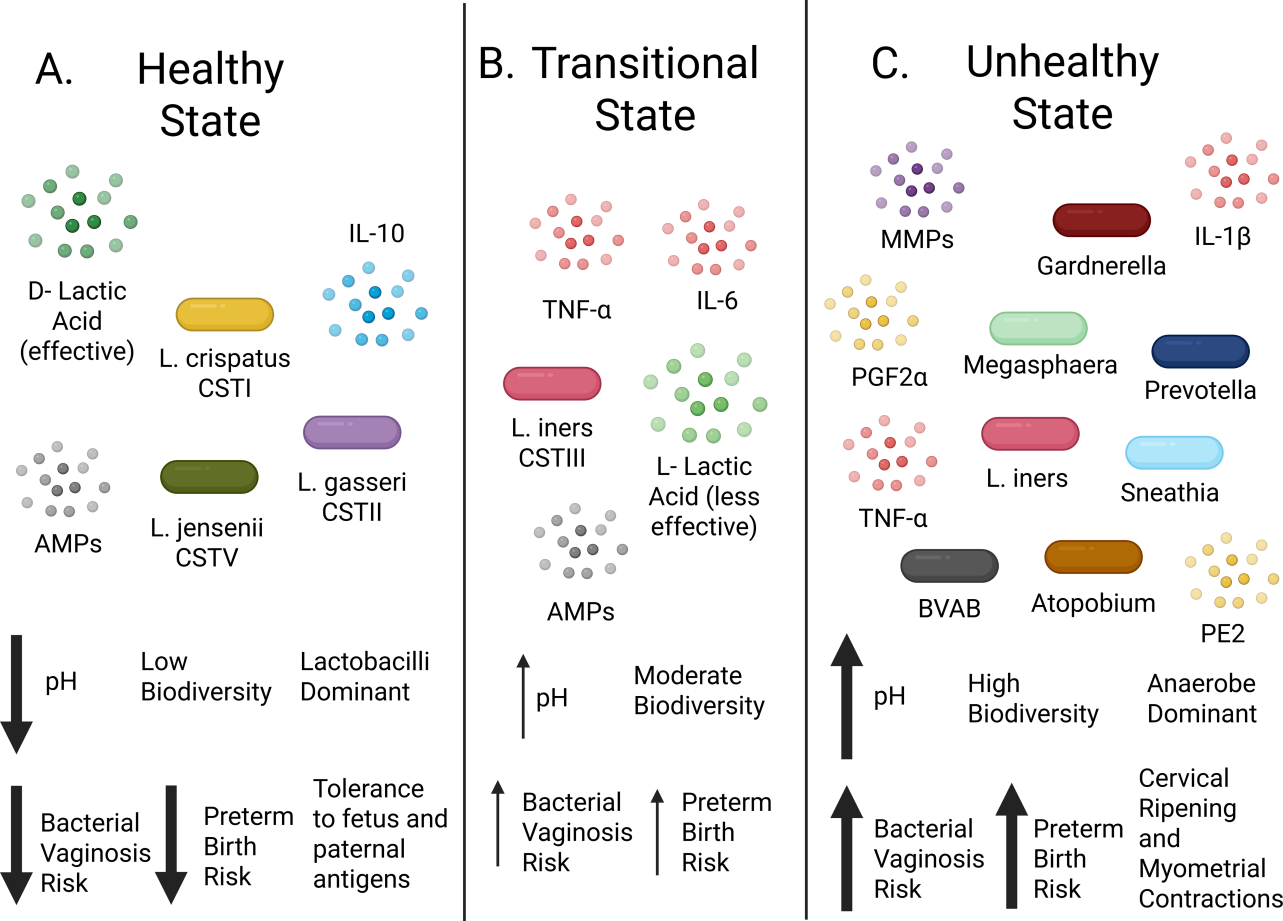
Proposed relation of Community State Types to vaginal health and birth outcome. **(A)** Representation of a healthy vaginal microenvironment dominated by lactobacilli species, *L. crispatus* (CSTI), *L. jensenii* (CSTV), *or L. gasseri* (CSTII). D-Lactic acid, with microbicidal activity, is highly effective at keeping vaginal pH low (~4-5). Antimicrobial peptides (AMPs) are secreted from epithelial cells of the female reproductive tract. Both lactic acid and AMPs discourage the growth of pathogenic bacteria and encourage low biodiversity. Detection of lactobacilli leads to the secretion of IL-10 from structural and immune cells to maintain an anti-inflammatory (immunotolerant state during pregnancy) microenvironment. **(B)** Representation of a transitional vaginal microenvironment dominated by *L. iners* (CSTIII). Biodiversity increases with the vaginal pH rising due to *L. iners* secreting a less effective microbicidal form of lactic acid (L-lactic acid). *L. iners* (and other microbes) induce the secretion of TNF-α and IL-6 from structural and immune cells promoting an inflammatory microenvironment. AMPs discourage the outgrowth of some vaginal microbes (anaerobes). **(C)** Representation of an unhealthy vaginal microenvironment dominated by anaerobes (CSTIV) with a low abundance of D- lactic acid secreting lactobacilli. Vaginal pH increases leading to the outgrowth of pathogenic bacteria taxa (anaerobes). Anaerobes prime structural and immune cells to secrete pro-inflammatory cytokines such as TNF-α and IL-1β. During pregnancy, increased levels of matrix metalloproteinases (MMPs- particularly 9), prostaglandins E2 (PE2) and F2α (PGF2α) in the decidua promote cervical ripening and myometrial contractions before labor. High biodiversity with an anerobic dominant microenvironment greatly increases the risk of Bacterial Vaginosis and preterm birth. Please see text for details and references (sections 2.2-2.4). Created in BioRender. Edwards, J. (2026) https://BioRender.com/xnxra96.

Lactobacilli are facultative anaerobic bacteria that ferment lactic acid. The main species of lactobacilli found in the FRT include *L. crispatus, L. iners, L. gasseri, and L. jensenii.* Co-dominance of any two of these lactobacilli species, however, is rarely observed (86). Lactobacilli are considered beneficial bacteria, as the secretion of lactic acid and hydrogen peroxide in the vagina ensures an acidic pH that is important for microbicidal activity against infectious pathogens (87–89). Importantly, lactic acid can occur in two isoforms, D- or L-lactic acid, and these isoforms differ in their microbicidal activity. Compared to *L. crispatus*, which predominantly produces the D-isomer, lactobacilli that predominantly produce L-lactic acid (like *L. iners*) are less effective in inhibiting the growth of opportunistic bacteria (90–92) (**Figures 3A, B**). In addition to maintaining an acidic milieu in the vagina, lactobacilli can also produce bacteriocins against other bacteria, thereby ensuring the persistence of lactobacilli dominance (88). In a few studies that isolated vaginal microbiota from healthy white women, in the event their vaginal microbiome lacked a dominance of Lactobacilli, other bacterial taxa, such as Gardnerella, Streptococcus, Enterococcus, Atopobium, Megasphaera, and Leptotrichia, were able to produce both D and L-lactic acid; however, L-lactic acid was dominant over D-lactic acid (91, 93). Furthermore, it has been suggested that vaginal epithelial cells can secrete lactic acid upon exposure to estrogen (94, 95). Human cells, however, only produce the less microbicidal form L-lactic acid (91, 96).

### 3.3 The contributions of bacterial vaginosis-associated bacteria to poor vaginal health

An imbalance of “healthy” lactobacilli to “bad” anaerobic vaginal bacteria is facilitating BV. Bacterial species that promote BV include predominantly bacteria belonging to CSTIV, such as Gardnerella (e.g., *G. vaginalis, G. piotii, G. pickettii, G. swidsinskii, G. greenwoodii*, and *G. leopoldii*) and the strictly anaerobe bacterial species Atopobium (e.g., *A. vaginae*), Mobiluncus (e.g., *M. curtisii, M. mulieris*), and Prevotella (e.g., *P. bivia, P. timonensis*, and *P. amnii*). CSTIV can be classified into three subgroups: subgroup A (BVAB1 dominant with a moderate abundance of *G. vaginalis*, *A. vaginae*, and *Prevotella* species), subgroup B (*G. vaginalis* dominant with a moderate abundance of BVAB1, *Prevotella*, and *A. vaginae*), and subgroup C (high biodiversity with an abundance of *Prevotella*, *Enterococcus*, *Bifidobacterium*, or*Staphylococcus* species (97). Subgroups A and B are highly linked to BV due to BVAB1, *G. vaginalis*, and *A. vaginae* in high abundance (98, 99). A high abundance of more than one of these species (e.g., *G. vaginalis* and *A. vaginae*) seems to further increase the risk for BV compared to the dominance of only one species (100–102). BV can increase the risk of obstetrical complications, pelvic inflammatory disease, and urogenital infections (103). Moreover, BV has been linked to an increased risk of miscarriage and preterm birth in pregnant women (103). The cause(s) leading to an imbalance of lactobacilli to BV-promoting bacteria are not well understood.

In a study including a cohort of (Black) South African women, CSTIII or CSTIV-dominated vaginal microbiomes were most prevalent. They observed that many of the BV-associated anaerobic bacteria (Gardnerella, Prevotella, Atopobium) promoted a more inflammatory milieu. Compared to CSTs I-III, bacteria of CSTIV induced higher levels of IL-1α, IL-1β, IL-8, and TNF-α, whereas IL-10 and IFN-γ levels did not differ (104). Similarly, Jespers et al. conducted a longitudinal study of vaginal microbiome composition in sub-Saharan African women (Kenya, South Africa, Rwanda) with incidental BV (n=40) and observed higher concentrations of *G. vaginalis*, *A. vaginae*, and *P. bivia* accompanied by increased concentrations of IL-1β and IL-12p70 (105). In addition, a higher “vaginal health score” was associated with decreased concentrations of IL-1α, IL-8, and IL-12p70. Prevotella species are also known to augment Th17 mucosal inflammation by activation of TLR2 (106). In addition, several BV-associated (e.g., Atopobium) species exert negative effects on vaginal epithelial barrier function by the production of mucins and antimicrobial peptides/defensins (107) (**Figure 3C**). Similarly, some CSTIII species (*L. iners*) have been associated with increased secretion of proinflammatory mediators and low metabolic functions by the vaginal mucosa and local immune cells (108) (**Figure 3B**). Furthermore, the transition of CSTIII to CSTIV and/or to a BV-promoting state is linked to biofilm formation, disruption of mucosal linings, and increased risk of acquiring sexually transmitted infections (STIs) (**Figures 3B, C**). The results of these studies support the direct interaction of vaginal microbiota with local immune cells and how downstream effects of these interactions alter the local milieu. Such interactions, and STIs in particular, could potentially impact pregnancy outcomes.

### 3.4 The contributions of vaginal microbiota to preterm birth

A US-based study (109) collected weekly vaginal swabs from a cohort of 40 pregnant women and determined changes in the vaginal microbiota throughout pregnancy. The vaginal microbiota of these women could be classified into the five CSTs earlier described by Ravel et al. (81). Despite differences in dominant CSTs, alpha and beta diversity of the vaginal microbiota appeared to be relatively stable in each of the pregnant women. Women with CSTIV exhibited the least stability in their vaginal flora, and transitions between CSTs were observed more frequently compared to women with CSTI, CSTII, CSTIII, or CSTV. Although the study consisted of only a relatively small number of women (n=40) and a majority of the participants were white (n=29), changes in vaginal microbiota over time in pregnancy and associations with term or preterm birth could be validated in an additional cohort of a larger sample size with more diversity in participants (109).

To establish a potential link between specific bacterial species and preterm birth, Anton et al. defined the immune profiles and barrier integrity of cervicovaginal epithelial cells (110). Vaginal, endocervical, and ectocervical cells were exposed *in vitro* to bacteria associated with either vaginal health or BV. The results demonstrated that live *G. vaginalis*, but not live *L. crispatus*, would induce cell death in vaginal, endocervical, and ectocervical epithelial cells. Bacteria-free supernatants had no effect on cell viability. Consistent with this finding, co-culture of cervicovaginal epithelial cells with live *G. vaginalis* increased cell permeability, an effect not observed in co-cultures with live *L. crispatus*. The exposure of vaginal, endocervical, and ectocervical epithelial cells to live *G. vaginalis* compared to live *L. crispatus* was associated with a higher secretion of several inflammatory mediators (e.g., IL-6, IL-8, eotaxin, GM-CSF, Macrophage Inflammatory Protein-1 alpha (MIP-1α), TNF-α, but a reduced induction of IL-3 and Platelet-derived Growth Factor AA (PDGF-AA) (110). Differences in cytokine induction were consistent with increased NF-kB activation by live *G. vaginalis* in HEK TLR2 reporter cells (110).

Co-culturing the various vaginal epithelial cells, instead of bacterial species, with cervicovaginal fluids of women with high *Gardnerella* species (*G.spp*) abundance (110). These results implied that the inflammatory milieu induced by *G. vaginalis* and other *G.spp*, at a minimum, is a contributing risk factor for preterm birth. Several other studies have reported an association between increased levels of IL1β and TNFα in women with preterm birth when compared to term birth (111–114). These cytokines upregulate MMPs, especially MMP-9, and prostaglandins (e.g., PGE2 and PGF2α) that contribute to cervical ripening and myometrial contractions before labor (**Figure 3C**). This suggests that, in contrast to lactobacilli, anaerobic bacteria induce proinflammatory cytokines and a rise in prostaglandins, which, in turn, could promote myometrial contractions and PTB.

In summary, one could conclude that a vaginal microbiome with low bacterial diversity (low Shannon index) and stable predominance of *Lactobacillus* (115), representative of CSTI, II, and V bacteria (81), appears to confer a low risk for PTB. A vaginal microbiome with high bacterial diversity (high Shannon index) and a stable predominance of *L. iners* or BV-associated bacteria, such as in CSTIII or IV (116, 117), increases the risk for PTB.

### 3.5 Vaginal microbiome compositions from different ethnic groups, BV, and preterm birth

As pointed out earlier (see section 2.1), populations of different ethnicities and races differ in the relative abundance of CSTs. In general, the vaginal microbiome in White/European women is dominated by CSTI (45%), whereas CSTIV (40%) is more common in Black/African women and in a subset of Hispanic women (81, 118). Healthy Black and Hispanic women with a vaginal microbiota dominated by CSTIV naturally have a higher vaginal pH (pH 4.7-5.0) compared to Asian or White women (average pH 4.5) (119). It is suggested that a higher vaginal pH facilitates the increased abundance of anaerobic bacteria and of lactobacilli that produce L-lactic acid (e.g., *L. iners*). An estimated 50% of women with CSTIV present with BV (120). Similar disproportionate rates are observed for BV when race and ethnicity of women are considered. Worldwide, BV affects 20% of women (121). Approximately 20-50% of sub-Saharan African women and 30% of U.S. women are affected by BV (120, 122). Categorized by race/ethnicity, an estimated 11% of Asian women, 22% of white women, 30% of Hispanic women, and 33% of African American/Black women of reproductive age have BV in the U.S (123). The percentage of pregnant women with BV ranges from 11%-50%, with higher prevalence in women from LMICs and women of African descent (120). The question, thus, arises whether women with distinct ethnic backgrounds face different risks for BV, preterm birth, or other adverse pregnancy outcomes. Indeed, some of the highest preterm birth rates occur in pregnant women living in the African region south of the Sahara (124). Even in the US, preterm birth occurs more often in Black women (14.7%) compared to White (9.5%), Asian (9.3%), or Hispanic (10.1%) women (125).

To address this important health problem, a sub-study of the United States Multi-Omic Microbiome Study-Pregnancy Initiative (MOMS-PI) in collaboration with the Global Alliance to Prevent Prematurity and Stillbirth (GAPPS) aimed to establish normal changes in the vaginal microbiome during pregnancy in women of different ethnicities and test whether specific host or microbiota factors were associated with preterm birth (118). The study population was selected to be enriched for pregnant women of African descent. Specifically, vaginal microbiota was defined in samples collected in early pregnancy (approximately 18 weeks of gestational age) for 90 women with term birth, of which 71 (78.9%) were of African descent, and 45 women with preterm birth, of which 35 (77.8%) were of African descent. The investigators identified 13 different community states, or “vagitypes”, based on the dominant bacteria in these women at the time of sample collection.

Women with term birth more often had a vaginal microbiota dominated by *L. crispatu*s, *L. delbrueckii*, or Streptococcus cluster 29 vagitypes. In contrast, women with preterm birth had a more diverse microbiota. In particular, women with preterm birth were more likely to have a higher abundance of BV-associated bacteria, including BV-Associated Bacterium 1 (BVAB1), *Candidatus Lachnocurva vaginae (*BVAB TM7-H1), *Sneathia amnii* (*S. amnii)*, *P. timonensis, P. buccalis*, and *Megasphaera type 1* (118). An association with preterm birth was also observed in women with Prevotella cluster 2, Streptococcus aglactiae-, or *Candidatus Mycoplasma girerdii*-dominant vagitypes. Data from metagenomics and metatranscriptomics analyses were generally concordant, and the results of metatranscriptomics further revealed that transcription of secretory proteins, which are associated with pathogenicity, was increased in bacterial species that were more highly associated with preterm birth. Lastly, longitudinal vaginal microbiome composition was assessed for women of African ancestry (AA) and women of European ancestry (EA) with term birth or preterm birth (118).

Overall, women with preterm birth presented with a high abundance of BV-associated bacterial species in their vaginal microbiome. Specifically, AA women had a higher abundance of BVAB1, *P. amnii*, *S. amnii*, and *G. vaginalis*. Furthermore, in women with term birth, an abundance of *L. crispatus* was more prevalent in EA women compared to AA women. Taken together, these findings suggest ethnic differences in distinct vaginal microbiota composition may modulate the risk for PTB.

### 3.6 Abnormal vaginal flora and the risk of ascending infections promoting preterm birth

It is estimated that between 20% to 40% of spontaneous preterm birth may be due to an infectious cause and resulting inflammation (8, 126, 127). Both maternal blood-borne infections and ascending vaginal infections can increase the risk for adverse pregnancy outcomes. BV, even when asymptomatic, is thought to enhance the possibility of pathogenic bacteria or their secreted products ascending and promoting preterm birth (128). Similarly, BV increases the risk of viral infections that can interfere with normal pregnancy progression. Below, we will discuss the effect of HIV on preterm birth and how HIV may be related to altered vaginal microbiota and macrophage function.

## 4 HIV, antiretroviral therapy, and preterm birth

### 4.1 The geographical overlap of HIV and preterm birth

The African region south of the Sahara not only accounts for a large percentage of preterm births but also represents the region with the highest burden of people living with HIV (PLWH). Globally, in 2024, there are approximately 41 million PLWH, of which 53% represent women and girls (129). Despite a marked decline in new infections, globally, 1.4 million new HIV infections were acquired in 2024, with women accounting for 45%. In African countries south of the Sahara (129), women account for 63% of all new infections. Every week, 4000 young girls and women (15 to 24 years) acquire HIV; among adolescents aged 15–19 years, girls accounted for 9 of 10 new HIV infections (130).

It is estimated that 1.3 million women and girls with HIV become pregnant each year. Current WHO guidelines recommend initiation of ART immediately upon diagnosis. These guidelines also apply to women who are pregnant, and HIV testing is generally performed during the first antenatal visit. Access to the various ART regimens is often less consistent in LMICs, and ART access is much lower in adolescent girls at high risk for pregnancy compared to adults. Considering that HIV is associated with immune dysfunction, it is crucial to understand the effect of HIV and ART on pregnancy. Importantly, HIV can be transmitted vertically from mother to child; thus, it can impact both maternal and infant morbidity and mortality. In a meta-analysis from 2015, the rate of preterm birth ranged from 5% to 73% in women living with HIV (WLWH) compared to women without HIV (2% to 32%) (131). Furthermore, preterm birth in WLWH was associated with a 5.2 to 8-fold increased risk of mortality compared to women without HIV (132, 133). Despite growing access to ART and better ART regimens that include integrase strand transfer inhibitors (INSTI), women with HIV continue to have a higher risk for adverse pregnancy outcomes (134). The biological mechanisms that enhance the odds of preterm birth in pregnant women with HIV, however, remain largely unknown. In this section, we will discuss how HIV-associated immune dysfunction and ART could potentially contribute to an enhanced risk for preterm birth in pregnant women with HIV.

### 4.2 HIV and immune activation

The primary receptor for HIV is the CD4 molecule, and the C-C motif chemokine receptor 5 (CCR5) serves as the main co-receptor. Other co-receptors, such as C-X-C chemokine receptor 4 (CXCR4), can be utilized by HIV. The CD4 protein is highly expressed on T_H_ cells, but CD4 can also be expressed on myeloid cells, although at a much lower density (135). Infection of myeloid cells is considered a relatively rare event by HIV strains that have evolved to infect CCR5-positive cells with low CD4 density (135, 136). The main route of HIV infection of macrophages is thought to be the engulfment of HIV-infected CD4 T cells (137, 138). Macrophages have a high expression of Tetherin, which traps the HIV virion at its cell membrane to prevent its release. In addition, macrophages increase the expression of SAM and HD domain-containing protein 1 (SAMHD1), which prevents viral replication and integration (139). Macrophages present HIV-derived peptides to CD4^+^ T cells, which supports the recruitment of CD8^+^ cytotoxic T cells (CTLs) to the site of infection, thus increasing the number of target cells HIV infect while HIV-infected macrophages and CD4^+^ T cells are killed by CTLs (140).

**Figure 4:**
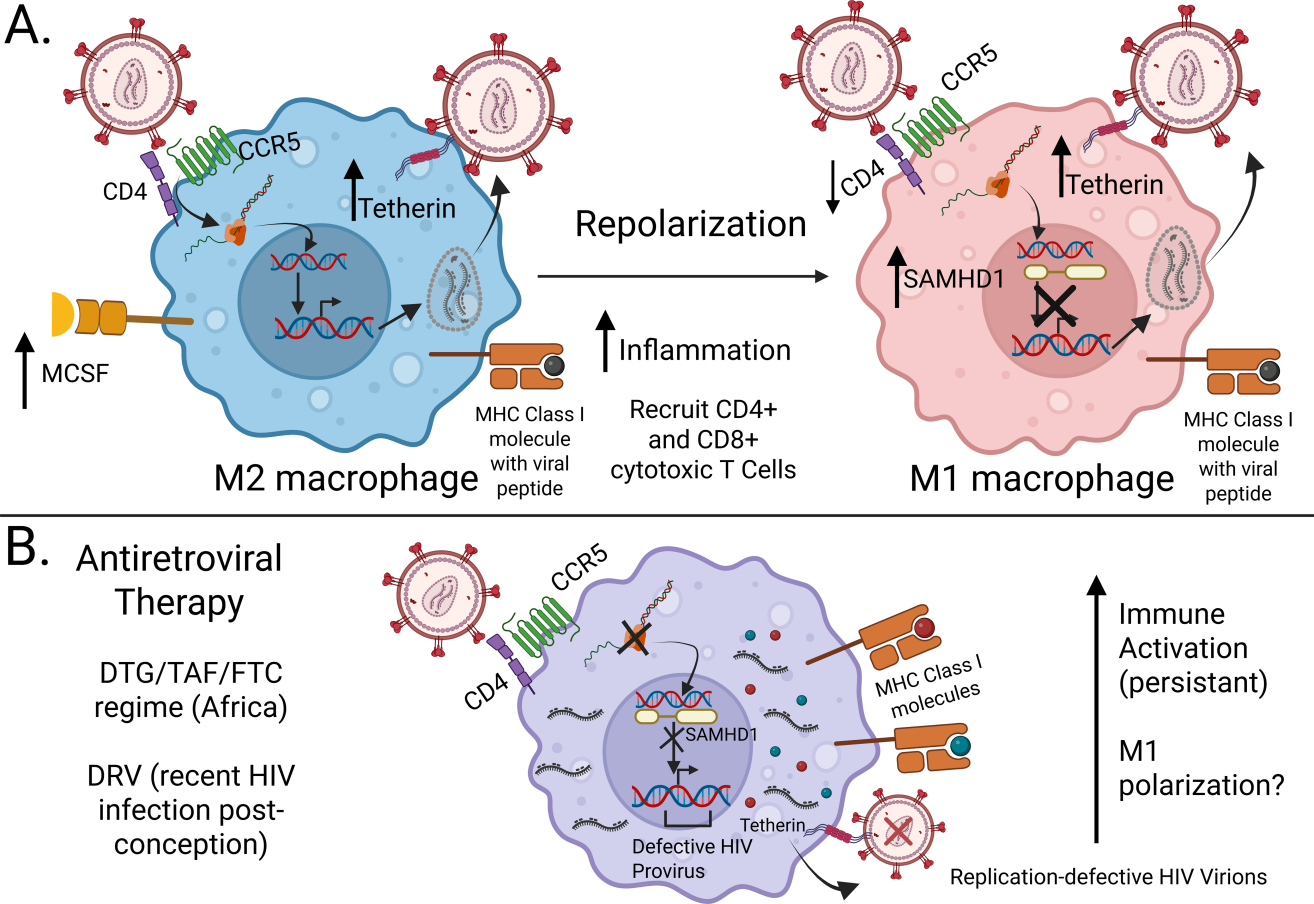
Proposed mechanism of HIV infection of macrophages with and without ART. **(A)** HIV initially binds to M2 macrophages (blue) via the CD4 receptor and CCR5 co-receptor. HIV RNA is released, reverse transcribed to DNA, integrated into the host genome, and assembled into a new virion. M2 macrophages increase the expression of Tetherin to prevent the release to HIV by binding the virion to the cell membrane (post- integration inhibition). Once M2 macrophages present HIV antigens to CD4^+^ T cells, the T cells will differentiate into CD4^+^ T_h_1 which in turn repolarize M2 macrophages to M1 macrophages (red). M1 macrophages increase the expression of SAMHD1, which inhibits viral replication and integration. Increased pro-inflammatory signals lead to the recruitment of CD4^+^ (highly susceptible to HIV infection) and CD8^+^ cytotoxic T cells (kill HIV infected macrophages and CD4^+^ T cells). **(B)** Antiretroviral therapy is very effective in decreasing viral load by preventing the production of new HIV virions. ART drugs target viral protein function (DRV), reverse transcription of HIV RNA (TAF and FTC) and integration of HIV DNA into host genome (DTG). Although infection of macrophages is less common compared to CD4^+^ T cells, macrophages (purple) could serve as a viral reservoir for defective HIV proviruses prior to ART initiation. Once cells become re-activated, these replication- defective HIV proviruses will produce replication-defective HIV virions. Nonetheless, viral proteins, such as Gag (Red) and Nef (Blue) can be presented by MHC class I molecules and then sensed by CD8^+^ cytotoxic T lymphocytes. The release of defective virions and the killing of viral protein expressing cells will contribute to immune activation. This can lead to increased M1 polarization, mucosal barrier dysfunction, and the translocation of microbes. Please see text for details and references (sections 3.2 and 3.3.2). Created in BioRender. Edwards, J. (2026) https://BioRender.com/b8mg6du.

Macrophage polarization also affects their susceptibility or resistance to HIV infection (141, 142). A review of macrophage relevance in HIV infection discussed that, in peripheral blood, high levels of M-CSF promote M2 polarization (139). Cassol et al. concluded that M2 macrophages restrict HIV-1 infection at a post-integration step and, thus, while HIV DNA can be integrated into the genome, virus release is inhibited (143). M1 macrophages restrict HIV-1 prior to integration and therefore have no restrictions on HIV virion release. Therefore, in M2 macrophages, Tetherin could be a factor in restricting HIV-1 infection at a post integration step whereas in M1 macrophages SAMDH1 could be a factor in restricting HIV-1 prior to integration. A summary of macrophage polarization during HIV infection is shown in **Figure 4A**. The contribution of HIV-infected macrophages to the viral reservoir is still subject to debate (143, 144). Thus, HIV directly targets APCs and CD4 T_H_ cells, important players of the innate and adaptive immune system, respectively, and, if untreated, will induce immunodeficiency due to severely impaired immune responses to the virus itself and to coinfecting pathogens.

HIV transmission occurs primarily through the sexual route. A study by Shen et al. demonstrated that vaginal and ectocervical dendritic cells in the human FRT from 15 minutes until 4 days post HIV exposure had the highest percentage of HIV infected cells. Macrophages were also shown to be permissive to HIV. These cells were responsible for the initial HIV transmission to other cells in the FRT (145). After 4 days, lymphocytes of the vaginal and ectocervical mucosa supported the highest level of HIV replication. Rapid depletion of CD4^+^ T cells at mucosal sites is a hallmark of HIV infection (146). Upon diagnosis and initiation of ART, CD4^+^ T cells will be reconstituted as viremia decreases (147). However, mucosal CD4^+^ T cell loss is generally irreversible (146). The loss of these mucosal CD4^+^ T cells, especially T_H_17 cells, results in reduced barrier function and microbial translocation (148–150). Although originally described for intestinal tissues, impaired epithelial cell integrity and microbial translocation also occur at other mucosal sites, including the female genital tract (151). These microbial products will be recognized by innate immune cells (e.g., neutrophils, macrophages, dendritic cells) and induce systemic immune activation in PLWH. In fact, monocytes and macrophages exhibit heightened immune activation and increased turnover in PLWH (152–154). Importantly, immune activation, although at reduced levels, persists even when viremia becomes fully suppressed by ART (155–157) (see Section 3.3.2). It is conceivable that heightened and persistent immune activation in pregnant women with HIV could interfere with the more immunoregulatory milieu required at the maternal-fetal interface. However, as HIV infection- only in some cases- leads to adverse pregnancy outcomes, factors other than immune activation likely contribute to the increased risk of preterm birth and other adverse pregnancy outcomes.

Maternal HIV infection during pregnancy, if undiagnosed, presents not only a serious health problem for the mother but may also result in vertical HIV transmission. A study by Pereira et al. revealed that virus-permissive cells fluctuate with gestational age, and infection rates tend to increase over time (158). Viruses reach permissible cells through the bloodstream or ascension from the lower FRT to the upper FRT, where maternal decidual and fetal placental cells reside (159). Interestingly, the decidua is more permissive to viral infection, whereas the placenta is more susceptible to bacterial infection based on the expression of PRRs (160). The severity of viral infection is also dependent on the maternal immune status, with high levels of phagocytic decidua dendritic cells and M2 macrophages resulting in a milder infection. Nonetheless, HIV-infected cells facilitate HIV transmission and promote immune activation that may have detrimental consequences for the fetus.

### 4.3 Antiretroviral therapy in pregnant women

#### 4.3.1 ART regimens

ART has become a cornerstone in HIV management and transformed the outcome of HIV from an almost always fatal disease to a chronic disease with near-normal life expectancy. The first anti-HIV drugs targeted the reverse transcriptase to block HIV replication. These initial single-drug regimens of nucleotide or non-nucleoside RT inhibitors (NRTI or NNRTIs) were associated with severe side effects, and HIV rapidly evolved resistance mutations (**Table 2**). The development of additional and safer NRTIs and NNRTIs, as well as drugs targeting the HIV protease and integrase, allowed the implementation of combination ART (cART) regimens that were not only more potent but also interfered with the ability of the virus to escape (161). The formulation of different antiretroviral drugs into a single tablet represented another milestone and was associated with improved adherence to ART. We have now entered the era of long-acting ARTs that can also be used as pre- and post-exposure prophylaxis. The benefits of ART are not limited to the increased life expectancy and quality of life for the person living with HIV but also translate (i) into reduced transmission rates due to faster and complete viral suppression, (ii) fewer double orphans, (iii) fewer vertical transmissions, and (iv) an overall higher economic return for society.

**Table 2:** Antiretroviral therapy drugs.

Function	Nucleotide RT inhibitors (NRTI)	Non-nucleoside RT inhibitors (NNRTI)	Integrase strand transfer inhibitors (INSTI)	Protease inhibitors (PI)
Drugs	*Tenofovir (TDF), *Tenofovir alafenamide (TAF), *Emtricitabine (FTC), *Abacavir (ABC),* Lamivudine (3TC), Zidovudine (AZT)	*Efavirenz (EFV), Nevirapine (NVP), Delavirdine (DLV), Etravirine (ETR), Rilpivirine (RPV), and Doravirine (DOR)	*Dolutegravir (DTG), Raltegravir (RAL), Elvitegravir (EVG), Bictegravir (BIC), Cabotegravir (CAB)	**Darunavir (DRV), Atazanavir (ATV), Amprenavir (APV), Fosamprenavir (F-APV), Indinavir (IDV), Lopinavir/ritonavir (LPV/r), Nelfinavir (NFV), Saquinavir (SQV), and Tipranavir (TPV).
cART regimens (pregnancy)	DTG/ABC/3TC DTG/TAF/FTC DTG/TDF/FTC EFV/TDF/FTC			

*Recommended for WLWH during Prenancy.

**DRV is recommnended for recently acquired post-conception HIV infection.

Yet, despite the introduction of highly active ARTs in the mid-1990s, WHO guidelines for ART use in pregnancy were only released in 2013 (162). There has been a concerted effort by the WHO and IMPAACT in the last few years to establish new guidelines for the safety testing of new ART regimens in pregnant women with HIV, and to include LMICs in these clinical trials (163, 164). New antiretroviral drugs may require dose adjustments due to marked alterations in blood volume and drug metabolism in pregnant women (165). A major challenge in the safety and toxicity assessment of ARVs in pregnant women is the relatively low frequency of adverse pregnancy outcomes. Extremely large cohort sizes are required to detect the impact of a specific antiretroviral drug or ART regimen on adverse pregnancy outcomes. Thus, there is a need for long-term surveillance studies in large pregnancy cohorts to detect safety signals (166–168).

As an example, shortly after the introduction of DTG, an initial report of the Tsepamo birth outcome study in Botswana reported that infants of pregnant women on DTG-containing regimens may have an increased risk of neural tube defects (169). However, a comprehensive analysis of birth outcomes in pregnant women from Botswana adhering to the DTG/TDF/FTC regimen as opposed to the EFV/TDF/FTC regimen showed no significant difference between preterm birth, low birth weight, neonate small body size, stillbirths, neonate deaths, or birth defects (170). Subsequent studies confirmed the safety of DTG in pregnant women with HIV, although DTG was associated with higher weight gain and, therefore, potential risk for hypertension (171). The WHO recommended DTG-based regimens for pregnant women with HIV in 2019 (172). The impact of DTG-including ART regimens on the risk for preterm birth remains controversial; a study in Nigeria found DTG to be without impact on preterm birth (173), whereas a larger meta-analysis found that both protease inhibitors (PIs) and INSTI regimens are associated with preterm birth and small for gestational age (SGA) (174). A comprehensive comparison of dual NRTI (TDF/FTC) regimens combined with various INSTIs in mice documented that, among the INSTIs, DTG had a better safety profile compared to BIC and CAB (175).

#### 4.3.2 ART, immune activation, and pregnancy

HIV infection can be characterized into two main phases (176, 177). Latent HIV infection is characterized by a phase of transcriptional silence and no production of virions. Persistent HIV infection is characterized by a phase of transcriptional activity with few virions produced. However, latent or persistent infection can be further characterized by the state of HIV provirus (176, 178). Replication-competent” proviruses can produce virions capable of replication whereas the “Replication-defective” proviruses produces virions incapable of replication (179).

Bruner et al. analyzed CD4^+^ T cells from 10 participants initiating ART during the chronic phase of infection and 8 participants during acute phase. Overall, they observed an accumulation of replication-defective HIV proviruses (180). In chronic HIV infection, defective proviruses were found in newly infected cells and reservoir cells. Furthermore, this accumulation of defective HIV proviruses also occurs rapidly during early HIV infection. Thus, a reservoir of defective proviruses is already present when ART is initiated during the acute phase HIV infection. During ART, CTLs mediate viral suppression (181) by recognizing cells harboring defective HIV proviruses if those cells produce viral proteins, such as Gag and Nef (182–184). Macrophages can also serve as viral reservoir and accumulate defective HIV proviruses- even during the use of ART (185, 186). Thereby, even defective HIV proviruses in macrophages and CD4+ T cells could contribute to persistent immune activation (187). A proposed mechanism for macrophages is illustrated in **Figure 4B**.

As stated above (Section 3.2), numerous studies documented that immune activation, although at a lower level, persists in PLWH once viremia is suppressed by ART. Shafiq et al. compared the immune profiles of pregnant Indian women without HIV to pregnant Indian WLWH but not on ART during the early 3^rd^ trimester and their infants 1 year post birth to assess maternal and newborn health (188). They found that higher levels of inflammatory cytokines (IL-17A and IL-1β) were correlated with an increased risk of preterm birth, low birth weight (IL-17A), or growth defects (IL-1β). Another study in India found that pregnant WLWH on ART had higher levels of the intestinal barrier dysfunction marker Intestinal Fatty Acid Binding Protein (I-FABP), and monocyte activation marker sCD14, and inflammatory cytokines IL-6 and TNF-α, when compared to pregnant women without HIV (189). Similar findings were reported by a study in France comparing monocyte activation and mucosal inflammation between WLWH on long-term ART treatment (median 6 years) to HIV uninfected women (190). WLWH had higher levels of sCD14, sCD163 (monocyte activation), sTNFRII, IL-17, IL-6, and I-FABP (inflammation). Whether certain antiretroviral drugs directly contribute to HIV-induced immune activation is not known.

#### 4.3.3 Potential influence of ART on the intestinal and vaginal microbiota

Disruption of mucosal barriers and resulting microbial translocation are the main drivers of immune activation in PLWH (Sections 2.2). This section will review the impact of diverse ART regimens on intestinal and vaginal microbiota.

Most studies assessing interactions of ART and microbiota include mainly male participants. A study of 69 Danish PLWH revealed that different ART regimens alter intestinal and oral microbiota uniquely. In comparison to people without HIV, all participants, independent of ART regimen, had increased levels of potentially pathogenic bacteria such as Succinivibrio, Megasphaera, Klebsiella, Escherichia-Shigella, and Ruminococcus gnavus. The ART regimen included a NRTI and a PI, but the third antiretroviral was either an INSTI (DTG or BIC) or a NNRTI. In PLWH who received the INSTI-containing regimen, “beneficial” *Faecalibacterium* and *Bifidobacterium* were enriched in the intestinal microbiota and “beneficial” *Veillonella* in the oral microbiota (191). Those who received a NNRTI instead of the INSTI experienced an increase in “beneficial” *Gordonibacter* and potentially “harmful” (pathogenic) *Megasphaera* and *Staphylococcus in the intestinal microbiota*, whereas the oral microbiota was enriched in “beneficial” Fusobacterium and *Alloprevotella.*

A longitudinal study in a Scandinavian cohort followed 16 viremic patients before and one year after ART initiation. Treatment with ZDV and EFV reduced Prevotella in the intestinal microbiota, and the antimicrobial activity of ZDF and EFV against Prevotella, as well as against *Bacteroides fragilis*, was confirmed *in vitro* (192). The effect of EFV on Prevotella was also observed in a transatlantic study involving the US, Uganda, and Botswana that assessed the fecal microbiome of 327 PLWH (193). Before ART, PLWH in Uganda, followed by Botswana, and then the US, presented with high alpha diversity and Prevotella abundance (193). HIV-associated alteration of microbial genes and pathways were mostly consistent across sites when PLWH without ART were compared to those on ART. However, the use of EFV, a NNRTI that is still widely used in Uganda and Botswana but not in the US, resulted in country-dependent gut microbiome alterations. The intestinal microbiome of PLWH from Uganda and Botswana who received EFV was depleted of Prevotella and exhibited signs of microbial network disruptions, reduced alpha diversity, and, as a result, more systemic inflammation. The dysregulation of gut ecology was thought to be mediated through ART-induced cross-inhibition of bacterial reverse transcriptases.

Another study followed 22 PLWH in Mali to determine the effect of TDF/3TC/EFV on the intestinal microbiome (194). Compared to the healthy control group, PLWH, both before ART and 12 months after ART initiation, had lower alpha diversity and low abundance of *Bacteroidetes (“beneficial”).* Conversely, the microbiota of PLWH pre-ART was characterized by an enrichment of inflammatory organisms such as *Escherichia (“pathogenic”)*. *Bacteroidetes*, *Clostridiales*, and *Ruminococcaceae* that are generally associated with gut health were more prevalent in the control group. Once ART was initiated, there was a decline in *Proteobacteria* and *Escherichia*. Overall, they observed a trend towards gut composition normalcy in the HIV group on ART for 12 months, but results lacked statistical significance due to small sample size. ART was also associated with negative alterations in metabolic pathways important for health, such as pyruvate fermentation and isobutanol metabolism; most likely due to a decrease in *Ruminococcus bromii* species.

Few studies explore the relationship between ART and vaginal microbiome composition changes in women, non-pregnant or pregnant. A study in Uganda followed non-pregnant women co-infected with HIV-1/HSV-2 (n=92) from 1-month pre-ART to 4–6 months after ART initiation. Although ART regimens varied among the participants, AZT/3TC/NVP was used by 84% (195). Based on their findings, CST classification of the vaginal microbiota was distinct from the common CST classification introduced above (Section 2.1): CST1 was characterized by *L. iners*, CST2 by Gardnerella, CST3 by Gardnerella and Prevotella, CST4 by *L. crispatus*, and CST5 was highly diverse. The study did not observe significant changes in the relative distribution of CSTs within the vaginal microbiota post-ART initiation (195). However, the bacterial load of Gardnerella decreased significantly after ART initiation when Gardnerella was the dominant species (CST2). Furthermore, CST4 (*L. crispatus*) had the lowest Shannon diversity (3.77) compared to other CSTs (>5.07). Lastly, immune reconstitution (CD4^+^ T cell count increase) following ART did not affect the composition of the vaginal microbiota.

In Malawi, pregnant WLWH (n=100) on ART (TDF/3TC/DTG) were compared to pregnant women without HIV (n=200) on PrEP (TDF and FTC). At enrollment, the majority of pregnant WLWH had a CSTIV-dominated vaginal microbiota with significantly higher levels of anaerobes (Gardnerella species, *S. amnii*, Prevotella species) when compared to non-HIV-pregnant women, who had a more even division between CSTIII or CSTIV-dominated vaginal microbiota with significantly higher levels of lactobacilli (196). In addition, WLWH had a higher Shannon α-diversity. However, the α-diversity and the abundance of anaerobic bacteria decreased after starting ART (4–6 weeks after enrollment). In contrast, α-diversity increased in non-HIV women taking PreP and increased or decreased a selection of lactobacilli and anaerobes. The authors also observed that despite the use of antiretroviral drugs, CSTIV remained associated with an increased risk for sPTB.

Combined, these studies suggest that ART can alter microbiota at different mucosal sites. While several studies point towards restoring “normal” microbiota post ART, there is also the potential of some ART regimens to disrupt the ecological balance.

#### 4.3.4 ART initiation pre-or post-conception

An important question that remains to be answered conclusively is whether the timing of ART initiation, pre- or post-conception, differently impacts pregnancy outcomes in women with HIV. Study designs to address this question have varied widely in inclusion and exclusion criteria, cohort sizes, and geographic locations, leading to conflicting results in terms of the timing of ART and its effect on the risk of preterm births and other adverse birth outcomes. A 2007 meta-analysis revealed a slightly increased risk for preterm delivery when women started ART pre-conception or during the 1^st^ trimester compared to women who initiated ART later in pregnancy (197). A later study that incorporated data from 19,189 mother-infant pairs to compare adverse birth outcomes based on pre- or post-conception ART (198) found that women with pre-conception ART were at higher risk of delivering preterm, very preterm, or infants with low birthweights when compared to women with post-conception ART. They found no significant difference, however, in the risk for SGA, stillbirth, or congenital anomalies. In contrast, a Canadian study reported a reduction of SGA cases in women who had initiated ART prior to conception (199).

Some of the controversial data can be explained by selection bias (200). In a 2018 study (200), ART initiation during pregnancy was further divided into early-term versus late-term post-conception ART to assess the impact of ART on preterm birth. However, their “naïve analysis” purposely excluded women who had preterm birth before ART was administered, while their “intent to treat” (ITT) analysis did not. They found that preconception ART was not associated with an increased risk of preterm birth in the ITT analysis, as rates of preterm birth were comparable to the post-conception ART group. In the naïve analysis, preconception ART was associated with preterm birth as the increased risk ratios coincided with the earlier gestational age of birth and preterm birth. Yet, as some women who were randomized to the group for late-term ART administration had already delivered preterm prior to ART start the selection bias had inflated the risk ratios for the preconception ART group while not accurately representing (or deflating) the risk ratios of the post-conception groups.

A more recent meta-analysis found that both preconception and antenatal initiation of ART were associated with an increased risk for preterm birth and SGA in women with HIV (201). However, this comparison was based on women without HIV as the comparator. A comparison of WLWH on ART versus WLWH but no ART revealed an even higher risk for SGA in women who were not on ART. Another study suggested that ART initiation in pregnancy is associated with a higher risk of preterm birth in Africa (202). The Promoting Maternal and Infant Survival Everywhere (PROMISE) 1077BF/1077FF study utilized a cohort of pregnant women with HIV who were randomized after delivery of their babies to either continue ART or stop ART and only restart ART upon becoming pregnant again (203). Women with preconception ART (or continued ART for subsequent pregnancy) had an increased risk of stillbirths, neonatal death, spontaneous abortion, and low birth weight compared to women who reinitiated ART. However, the risk of preterm birth was comparable between the two groups (203). These findings were similar to previously reported studies by Kourtis (197) and Uthman (198).

Overall, the question of whether the timing of ART initiation, pre- or post-conception, differently impacts adverse pregnancy outcomes in WLWH remains to be answered conclusively. Furthermore, it should be noted that there are only a few studies that examine whether the timing of ART initiation impacts pregnancy outcomes since the introduction of DTG-based ART regimens in pregnancy. Selection bias, differences in defining the time of ART initiation relative to conception, and distinct ART regimens in studies prevent establishing a definite link between pre- or postconception ART initiation and a higher risk for preterm birth. For similar reasons, the notion of associations between specific adverse pregnancy outcomes with specific ART regimens remains controversial (see Section 4).

## 5 Potential interactions of risk factors for preterm birth

### 5.1 Macrophages, vaginal microbiota, HIV/ART, and preterm birth

The current review so far delineates how altered macrophage function, changes in vaginal microbiota, and HIV/ART as individual factors can modulate pregnancy outcomes, especially preterm birth. This section will outline potential interactions between macrophages, vaginal microbiota, and HIV/ART and discuss whether these biological interactions may enhance the risk of preterm birth more than the risk presented by each of the factors alone.

The growth and development of the allogenic fetus is dependent on a tolerogenic milieu at the maternal-fetal interface. Lactobacilli produce short-chain fatty acids (SCFA) that induce the differentiation of regulatory T cells (204, 205) that support tolerance. However, when the vaginal microbiota is dominated by anaerobic strains (84, 206), an inflammatory milieu is promoted (84, 207). The presence of some anaerobe bacteria *(e.g., M. curtsii/muleris)* has also been associated with low levels of beta defensin 2 (BD2), a peptide with anti-HIV activity (208). WLWH exhibits additional microbiome perturbations due to microbial translocation (148, 209, 210), which in turn has been directly linked to macrophage turnover in HIV infection (211). In pregnancy, macrophages are important in the defense against ascending bacterial and viral infections, especially those related to chorioamnionitis (159, 212–214).

### 5.2 “Dual-Hit Model” for increased risk of sPTB in sub-Saharan African WLWH

While the role of macrophages in preterm birth remains poorly understood, it is conceivable that the perturbations (diversity, species abundance, secreted metabolites) in vaginal microbiota are sensed by macrophages and incite inflammatory responses that will promote preterm birth. In fact, various cells of the maternal-fetal interface, including trophoblasts, macrophages (maternal decidual and fetal Hofbauer cells), and placental fibroblasts, can express PRRs. However, PRR expressions are dynamic and regulated by environmental stimuli, which in turn activate specific immune responses. The knowledge of TLR expression is primarily based on immunohistochemistry data from placental tissues collected after birth, and isolation of primary decidual cells and amniotic fluid. Multiple studies revealed that TLRs 1–6 are prominent in decidual cells and that soluble forms of TLR2 and TLR4 can be found in the amniotic fluid (215, 216). Transcriptional profiling revealed that PRRs (in particular TLR4 and TLR2) on dendritic cells and macrophages sense CSTIV bacterial products. The subsequent induction of various signaling pathways then promotes transcription of proinflammatory cytokines and chemoattractants (105). In the placenta, the expression of TLR2 was prominent on endothelial cells and fetal macrophages called Hofbauer cells (HBC), whereas TLR4 expression was most prominent on syncytiotrophoblasts and fibroblasts (217, 218).

**Figure 5:**
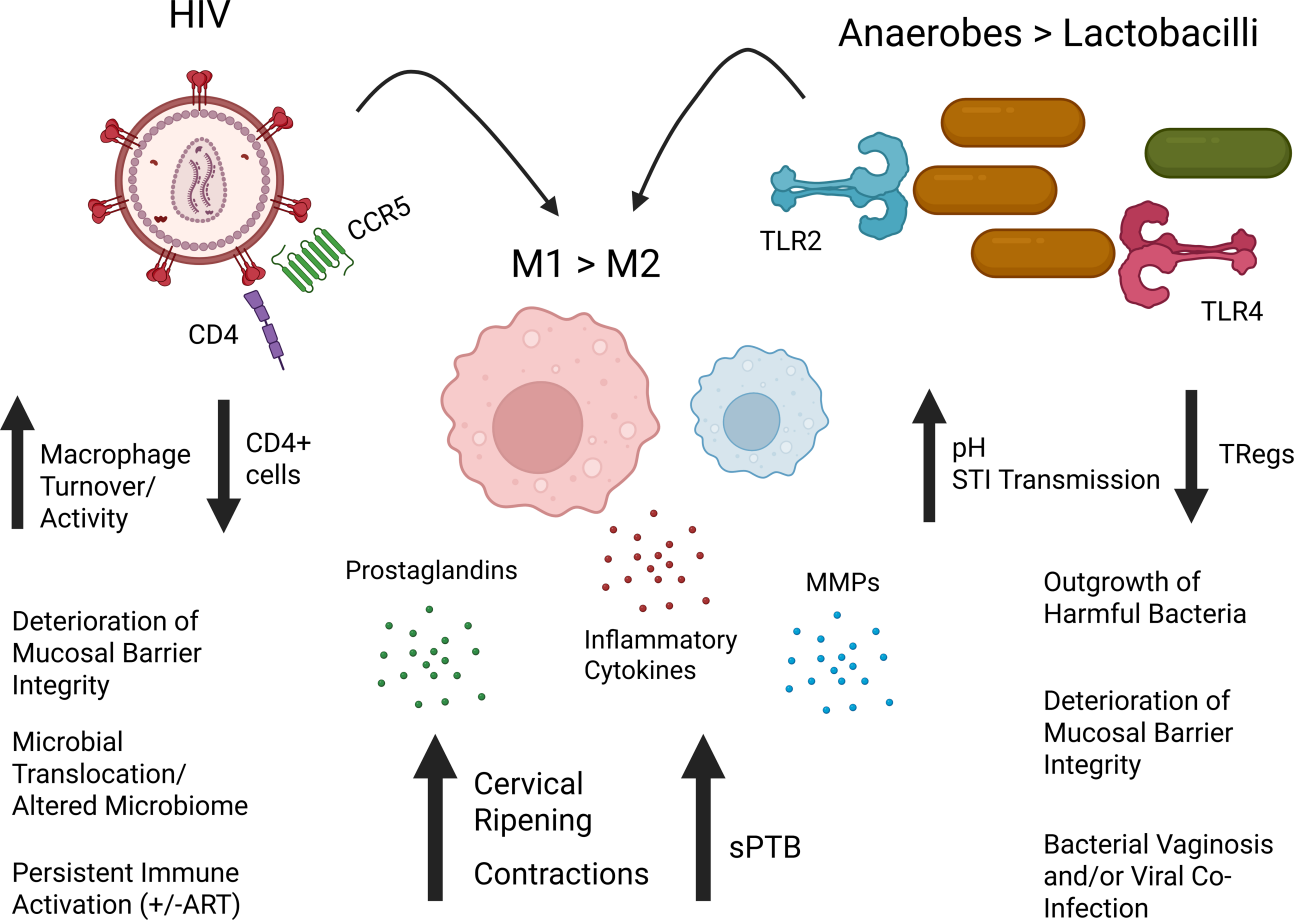
Proposed “Dual-Hit” Model of sPTB in sub-Saharan African WLWH. We hypothesize that HIV infection and anaerobic- rich microbiomes commonly found in sub-Saharan African women incite inflammation. The alteration of the vaginal microbiome by HIV infection will cause inflammation-driven deterioration of mucosal barrier integrity and subsequent microbial translocation. Anaerobes (brown), primarily sensed through TLR2 and 4, support the outgrowth of harmful bacteria by rising the pH, contribute to inflammation-driven deterioration of mucosal barriers, and increase the risk of BV and STI transmission- leading to co-infections. Overall, during pregnancy, persistent immune activation, despite the use of ART, will favor a dominance of M1 macrophages (red) over M2 macrophages (blue) establishing an inflammatory environment. In response, cells of the Female Reproductive Tract will produce prostaglandins and metalloproteinases (MMPs) to facilitate cervical ripening and myometrial contractions which will initiate spontaneous labor. Thus, WLWH and an anaerobic dominant vaginal microbiome will have an increased risk of sPTB. STI, Sexually Transmitted Infection. Created in BioRender. Edwards, J. (2026) https://BioRender.com/cg187sh.

Persistent immune activation in PLWH, even when viremia is fully suppressed, has been attributed to disrupted mucosal barriers, microbial translocation (148, 219), and changes in microbiome composition (149, 155, 220, 221). These bacteria and their bacterial products can be sensed by PRRs on macrophages. *In vitro* studies with human vaginal and amnion epithelial cells demonstrated increased responses to TLR2 and TLR2/6 (bacterial) agonists after initial stimulation with TLR3 (viral) agonists (222). In contrast, a dampened response to LPS, a TLR4 ligand, has been identified as an important pathway in pregnancy maintenance and term delivery (223). Thus, it is feasible that in WLWH, the anaerobic nature of the vaginal microbiome and HIV-associated immune activation converge to provide a “dual hit”. As a result, macrophages become activated and functionally polarize towards M1, thereby disturbing the tolerogenic state in mid-pregnancy and enhancing the risk for preterm birth (**Figure 5**).

In the Zambian Preterm Birth Prevention Study (ZAPPS)- consistent with data in Malawian women (206)- the vaginal microbiome of pregnant women, independent of birth outcome, was dominated by *G. vaginalis* and other anaerobic species (84). However, among pregnant women of the ZAPPS cohort, WLWH had the highest prevalence of anaerobic bacterial strains and the highest Shannon index (84). Applying metagenomics, Dr. Ravel identified a novel *Gardnerella* sp*ecies* associated with preterm delivery. *G. vaginalis*, a known *G. ssp* linked to PTB (116, 117)*. G. ssp* has been associated with reduced epithelial integrity (224), further facilitating “leaking” of microbes and potential macrophage activation. Data from the ZAPPS cohort demonstrated an increased risk (18.3%) of preterm birth in WLWH compared to women without HIV (14.0%). The HIV-associated risk was even more amplified when the analysis was limited to spontaneous preterm birth (sPTB). sPTB occurred in 8.7% of women without HIV compared to 14.3% and 15.0% WLWH on pre- or post-conceptional ART, respectively (225). The latter data further implied that ART and/or timing of ART initiation do not mitigate the risk for sPTB (200, 226). When HIV status was disregarded, and the vaginal microbiome metagenomic clusters (mgClust) were stratified based on birth outcome, the mgClust associated with sPTB included *G. vaginalis, L. iners, A. vaginae*, Prevotella species, and BVAB1. This data is consistent with the bacterial species that were more prevalent in the vaginal microbiota of AA women with preterm birth identified in the United States MOMS-PI study (section 2.5).

The sPTB risk phenotype of the vaginal microbiome might need to be further defined for women in sub-Saharan Africa where (s)PTB is highly prevalent, e.g. by identifying the specific bacterial strains augmenting sPTB risk and/or inclusion of additional risk factors. In contrast to lactobacilli, anaerobic bacteria induce proinflammatory cytokines (88, 89, 114, 227) and induce a rise in prostaglandins, which, in turn, can promote myometrial contractions and sPTB. The ZAPPS data supports such a link between an anaerobe-dominated vaginal microbiome, local inflammation, HIV/ART, and sPTB (84).

## 6 Conclusions, challenges, and opportunities

This review focuses on select risk factors of sPTB such as macrophage function, vaginal microbiota, and HIV/ART in the specific population of sub-Saharan African women living with HIV. Preterm birth remains a heavy burden in LMICs in Africa and Southeast Asia, and sub-Saharan Africa continues to have a high burden of HIV, especially among women. HIV is a recognized risk factor of preterm birth. African women often also present with a more anaerobe, CSTIV- dominant vaginal microbiota that has been associated with preterm birth independent of HIV status. Furthermore, there is growing evidence of shared biological risk factors for preterm birth among women of African descent (sub-Saharan African, Afro-Caribbean, Black American), and these factors are not necessarily related to geographical, socio-economic, or healthcare infrastructure differences.

Considering the multitude of factors that can contribute to sPTB, better-designed studies are needed to conclusively identify biological mechanisms that promote sPTB. Models, including the above-described “dual-hit model”, remain speculative and require confirmation. Yet pregnancy cohort studies to define mechanisms of adverse birth outcomes are challenging due to limited access to human tissue specimens of the maternal-fetal interface. Animal models of pregnancy have varying degrees of translational value. Large-scale cohort studies, ideally starting in the pre-conception period, with longitudinal follow-up, carefully selected specimen collection, multipronged analysis tools (e.g., multi-omics), and integration of demographic, socio-economic, and clinical experimental data are needed to ascertain biological pathways with a causative link to sPTB. **Box 1** lists some of the challenges and opportunities regarding the goal of preventing sPTB or reducing the severity of sPTB outcomes for maternal and infant health.

Box 1 **Challenges, limitations, and opportunities in the quest to prevent preterm birth.**

Challenges and limitations:

ο Limited access to relevant tissue specimens from the maternal-fetal interface.
ο Lack of animal models with high translational value to human pregnancy.
ο Highly variable study design of human pregnancy cohorts.
   -time-span and follow up.
   -specimen types and collection time points.
ο Cohort size and case numbers.
ο Consideration of site-specific factors (e.g., geographical location, HIC vs LMICs; infrastructure; socio-economic factors).
ο Cohort initiation in pre-conception period.
ο Data validation in an independent cohort.

Opportunities:

ο Develop improved organoid systems to study biological processes at the maternal-fetal interface.
ο Large-scale pregnancy cohorts with structured longitudinal follow up that includes clinical exams and relevant and diverse specimen collection.
ο Oversight of a well-established biorepository of specimens from pregnant women with clinically defined pregnancy outcomes.
ο Adaptation of clinical devices (e.g., ultrasound equipment) that is portable for use in rural areas and regions with limited healthcare infrastructure.
ο Application of novel multi-omics technologies to define differences in biological parameters throughout pregnancy in women with or without sPTB.
ο Utilization of bioinformatics to integrate different data types.
ο National and international collaborations and access to biorepositories and databases.
ο Collaboration of clinicians, nurses, midwives and researchers combined with educational outreach activities to communities.

In conclusion, the identification of specific biological pathways will inform the development of early prevention and intervention strategies for populations at high risk of preterm birth, such as women living with HIV and women of African descent.
